# Lateral rectus abdominis approach fixation of a high-energy both-column acetabular fracture after total hip arthroplasty with retention of a stable acetabular cup: a case report

**DOI:** 10.3389/fsurg.2026.1794712

**Published:** 2026-04-23

**Authors:** Xiangyu Zong, Qicai Li, Chunpu Li, Hongtao Ge, Xuemei Yang, Yingze Zhang, Tianrui Wang

**Affiliations:** 1 Knee Preservation Center, The Affiliated Hospital of Qingdao University, Qingdao, Shandong, China; 2 Department of Joint Surgery, The Affiliated Hospital of Qingdao University, Qingdao, Shandong, China; 3 Department of Sports Medicine, Qingdao Hospital, University of Health and Rehabilitation Sciences (Qingdao Municipal Hospital), Qingdao, Shandong, China; 4 Department of Spine Surgery, The Affiliated Hospital of Qingdao University, Qingdao, Shandong, China; 5 Department of Operating Room, Qilu Hospital (Qingdao), Cheeloo College of Medicine, Shandong University, Qingdao, Shandong, China; 6 Department of Trauma Emergency Center, The Third Hospital of Hebei Medical University, Hebei Shijiazhuang, China

**Keywords:** acetabular fracture, cup preservation, lateral rectus abdominis approach, periprosthetic acetabular fracture, total hip arthroplasty

## Abstract

**Background:**

Acetabular fractures are intra-articular injuries with complex anatomy and demanding reduction requirements. Traumatic periprosthetic acetabular fractures after total hip arthroplasty (THA) are rare and particularly challenging because treatment must simultaneously address fracture stability and acetabular component stability.

**Case presentation:**

A 72-year-old woman sustained a high-energy road-traffic injury from an outside vehicle 10 years after left THA. Computed tomography (CT) demonstrated a comminuted both-column acetabular fracture with medial displacement of the quadrilateral surface and compromised periacetabular bone continuity, raising concern for cup instability. Open reduction and internal fixation (ORIF) was performed in the supine position through a lateral rectus abdominis approach (LRAA). Intraoperative direct visualization and fluoroscopy confirmed a well-fixed, osseointegrated acetabular cup, which was therefore retained. The anterior and posterior columns and quadrilateral surface were reconstructed using two contoured reconstruction plates, with careful screw trajectory planning to avoid the cup.

**Conclusion:**

For traumatic periprosthetic both-column acetabular fractures after THA, intraoperative assessment of acetabular component stability is pivotal. When the cup is stable, LRAA can provide direct intrapelvic exposure enabling anatomic reduction and robust buttress fixation of the quadrilateral surface while avoiding revision arthroplasty.

## 1 Introduction

Acetabular fractures are relatively uncommon; however, they impose a substantial burden of disability. Population-based data suggest that the incidence of pelvic and acetabular fractures has increased over time, reflecting both high-energy trauma in younger patients and low-energy fragility mechanisms in older adults (1–4). As intra-articular injuries, acetabular fractures frequently involve the anterior and posterior columns as well as the quadrilateral surface. The quality of reduction is a key determinant of long-term hip survivorship and the risk of post-traumatic osteoarthritis (5, 6).

With the widespread use of THA for femoral neck fractures, osteonecrosis of the femoral head, and end-stage hip disease, the postoperative population continues to expand. Compared with femoral-sided periprosthetic fractures, traumatic PPAFs are even less common, yet they are associated with high complication and reoperation rates (7). Inadequate management can markedly compromise hip function and predispose patients to severe complications, including component loosening and dislocation, which may necessitate revision surgery and are linked to an increased mortality risk (8). The clinical complexity of PPAFs stems from their nature as a coupled injury involving an intra-articular fracture and an implanted mechanical construct. Following THA, wear-related changes and acetabular cup implantation further alter local anatomy and the mechanical environment, resulting in more complex fracture patterns and greater difficulty in achieving reduction and stable fixation, thereby posing major therapeutic challenges (9). Management requires simultaneous assessment of fracture morphology, bone stock, and cup stability to guide the choice among isolated internal fixation, fixation combined with revision, or staged procedures (10–12). To date, reports of both-column acetabular fractures after THA in which the acetabular component is ultimately retained remain limited.

We report a case of a patient who sustained an acetabular both-column fracture due to a collision with an outside vehicle while walking, 10 years after THA. Intraoperatively, the acetabular cup was confirmed to be stable; reduction and fixation of both columns and the quadrilateral plate were accomplished via the lateral rectus abdominis approach, and the acetabular component was successfully preserved. This report summarizes key considerations for assessment, surgical strategy, and approach selection in this rare and complex injury pattern, with the aim of informing management of similar cases.

## 2 Case report

### 2.1 Patient information

A 72-year-old woman was admitted with a 1-day history of left hip pain and inability to mobilize after a motor vehicle collision. She was initially evaluated at an outside hospital and transferred to our institution the following day. Her medical history was notable for hypertension for more than 4 years, controlled with oral antihypertensive therapy (nifedipine, three times daily), with satisfactory blood pressure control. Ten years earlier, she had undergone left THA for a femoral neck fracture and had been able to ambulate independently thereafter. She also reported a history of poliomyelitis in childhood, with residual limp and ipsilateral asymmetry in muscle strength and soft-tissue development. The patient had an unremarkable personal history. There was no family history of genetic diseases or psychiatric disorders.

On physical examination, the left hip was painful with markedly restricted range of motion, and swelling with ecchymosis was present over the left gluteal region. The left lower limb was approximately 1 cm shorter than the right. Longitudinal percussion elicited tenderness along the left tibia. The patient declined passive range-of-motion testing due to pain. Skin temperature and sensation were intact in the left lower limb. Left ankle motion was more limited than on the contralateral side, and hypoplasia of the Achilles tendon was palpated posteriorly. The dorsalis pedis pulse was palpable.

**Figure 1:**
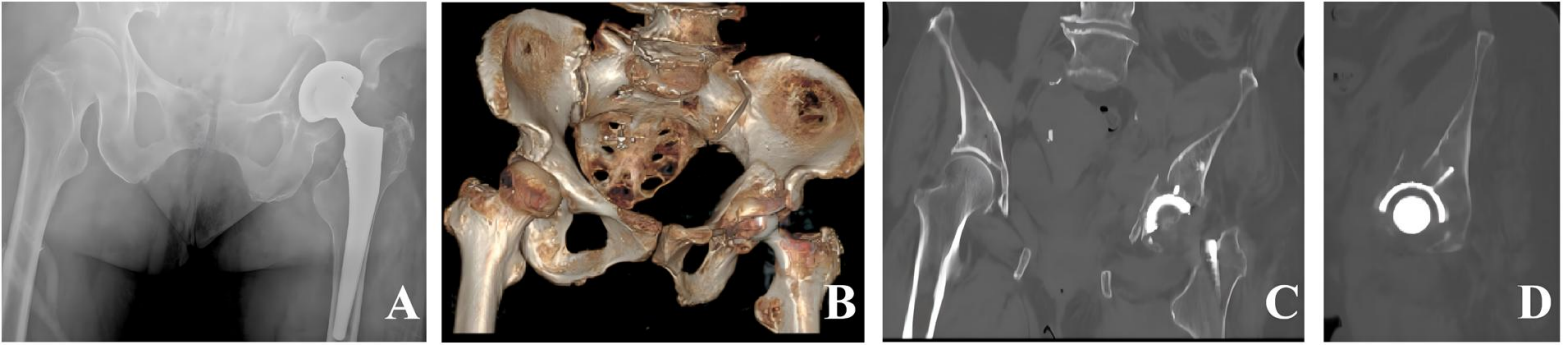
Preoperative radiographs and CT. **(A)** preoperative anteroposterior x-ray images of the pelvis. **(B)** Three-dimensional CT reconstruction. **(C)** Coronal CT image. **(D)** Sagittal CT image.

Imaging studies included an anteroposterior pelvic radiograph, which demonstrated disruption of the left acetabular contour with displaced fracture fragments; the prior THA components remained *in situ* (Figure 1A). CT with three-dimensional reconstruction revealed a comminuted both-column acetabular fracture with medial displacement of the quadrilateral plate and loss of bony continuity around the acetabular cup; preoperative imaging suggested possible acetabular component instability (Figures 1B–D).

### 2.2 Diagnostic assessment and reasoning

Differential diagnosis at presentation included: (1) hip dislocation; (2) traumatic acetabular component loosening or dissociation; (3) femoral periprosthetic fracture.

Preoperative radiographs raised concern for possible component instability due to medialization and fracture displacement. This uncertainty was resolved intraoperatively by direct inspection of the interface through the fracture lines and dynamic fluoroscopic observation during reduction and fixation, which demonstrated a well-integrated cup without micromotion or positional change.

Prognostic considerations included nonunion, secondary component loosening or migration, and the potential need for delayed revision if instability developed. Given these risks, the treatment goals were anatomic reduction, medial buttress reconstruction, and structured imaging follow-up including CT to confirm fracture union. The timeline of the treatment process is presented in Table 1.

**Table 1:** Timeline of the treatment process.

Time point	Key events	Findings/Actions
Day 0 (Injury) −1	Struck by a motor vehicle while walking	Severe hip pain; inability to bear weight; presented to a local hospital immediately after injury; transferred to our hospital the following day.
Day 1–2	Initial imaging	Radiographs + CT: both-column acetabular fracture with quadrilateral surface medialization and a risk of acetabular cup displacement.
Day 2–3	Preoperative planning	Plan for ORIF with intent to retain cup if stable
Day 4	Surgery	LRAA exposure; cup stability assessment; anatomic reduction; plate buttress fixation
Post-op week 0–6	Rehabilitation	Bed rest; joint and muscle functional rehabilitation; DVT prophylaxis; wound care; serial radiographs
3 months	Follow-up visit	Protected weight bearing; no migration; progressive healing on radiographs
6 months	Follow-up visit	CT confirmed union; stable component; full weight bearing

### 2.3 Surgical technique

**Figure 2:**
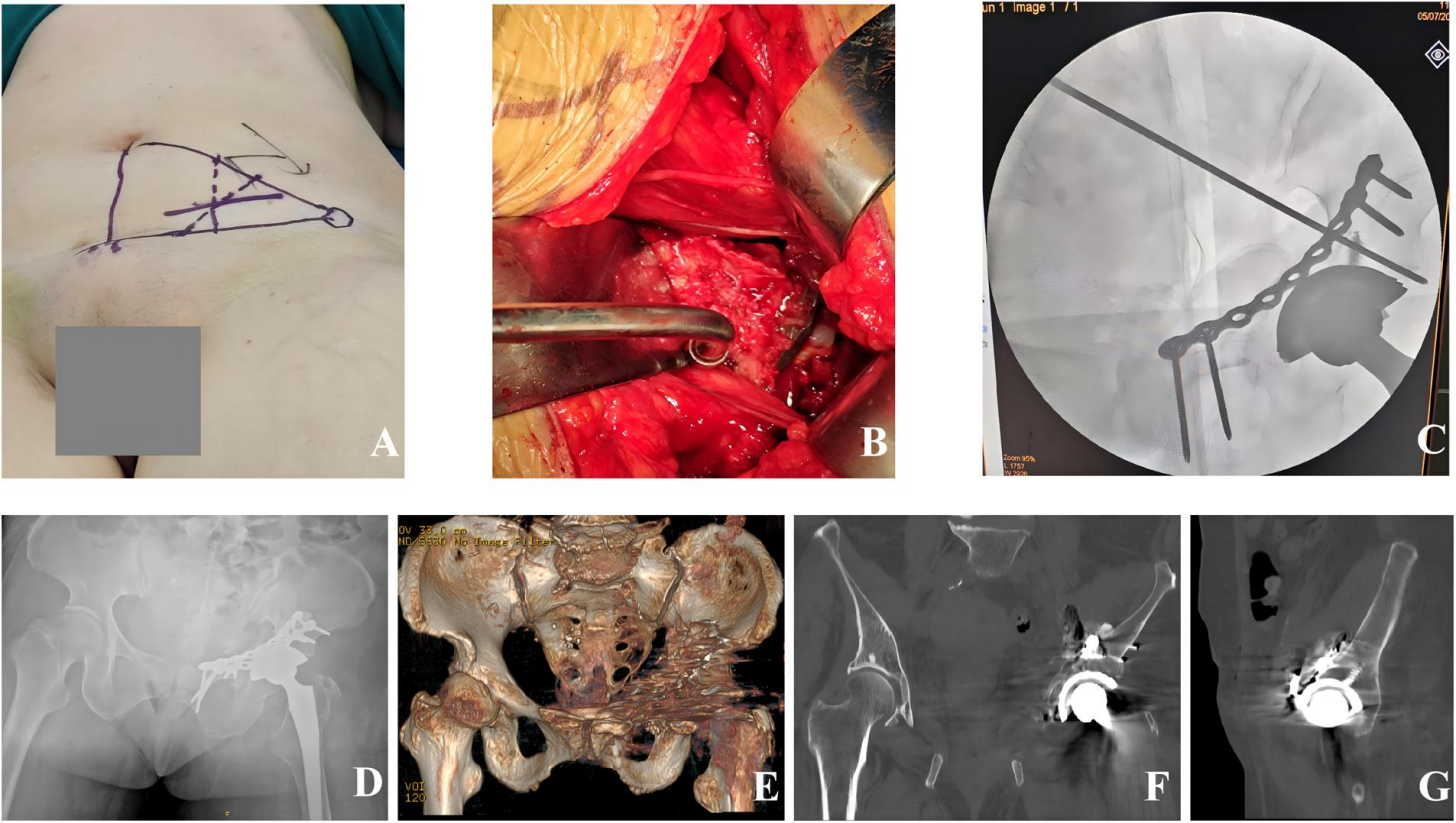
Intraoperative and postoperative images. **(A)** Patient positioning and incision for the LRAA. **(B)** Direct visualization of the acetabular fracture and acetabular cup. **(C)** Fluoroscopic confirmation after reduction and fixation. **(D)** Postoperative imaging of anteroposterior pelvic radiograph. **(E)** Postoperative imaging of three-dimensional CT reconstruction. **(F)** Postoperative imaging of coronal CT image. **(G)** Postoperative imaging of sagittal CT image.

After induction of general anesthesia, the patient was positioned supine (Figure 2A). A skin incision of approximately 7 cm was made along the interval defined by the junction of the lateral two-thirds of the line from the left anterior superior iliac spine to the umbilicus and the medial two-thirds of the line from the ASIS to the pubic symphysis. The skin, subcutaneous tissue, and external oblique aponeurosis were incised in layers. The internal oblique and transversus abdominis muscles were bluntly split to enter the extraperitoneal space. The peritoneum was retracted medially, and the superficial fat was bluntly cleared with a periosteal elevator to expose the iliopsoas muscle, the external iliac vessels, and the femoral nerve. The neurovascular bundle was not dissected separately; instead, it was mobilized *en bloc* and retracted medially. Through the interval between the iliopsoas and the neurovascular bundle, the anterior wall, anterior column, quadrilateral surface, and the periprosthetic region were accessed. Hematoma and interposed soft tissue at the fracture site were debrided. Intraoperatively, displacement of the quadrilateral plate was noted, and the acetabular cup was directly visualized (Figure 2B). The neurovascular bundle was looped with a vessel loop and, together with an “S”-shaped retractor, used to facilitate exposure while protecting the obturator nerve. The corona mortis was identified and ligated, and the iliopectineal fascia was incised.

Subsequently, we performed intraoperative assessment of acetabular cup stability using low-risk and practical criteria. Specifically, we directly inspected the bone–implant interface through the fracture lines for gross separation or fibrous tissue, assessed for visible micromotion of the cup relative to the host bone during gentle manipulation of the reduced columns and quadrilateral surface, monitored the cup under fluoroscopy during reduction and fixation to exclude toggling, change in inclination/anteversion, or progressive medialization, and confirmed that buttress fixation could be achieved without violating the cup or compromising its osseointegrated surface. No motion, interface disruption, or positional change was observed, supporting cup retention.

Key fragments and the anterior column were reduced using a ball-spike pusher and reduction forceps, followed by temporary Kirschner-wire fixation. A contoured plate was then applied to stabilize the anterior column (Figure 2C). Subsequently, a reconstruction plate was placed along the posterior column, with care taken to avoid screw penetration into the acetabular cup. After fluoroscopic confirmation of satisfactory reduction, appropriate implant positioning, and component stability, the wound was irrigated with normal saline and closed in layers. Allogeneic bone grafting was performed around the fracture site, followed by layered closure.

### 2.4 Follow-up and outcomes

**Figure 3:**
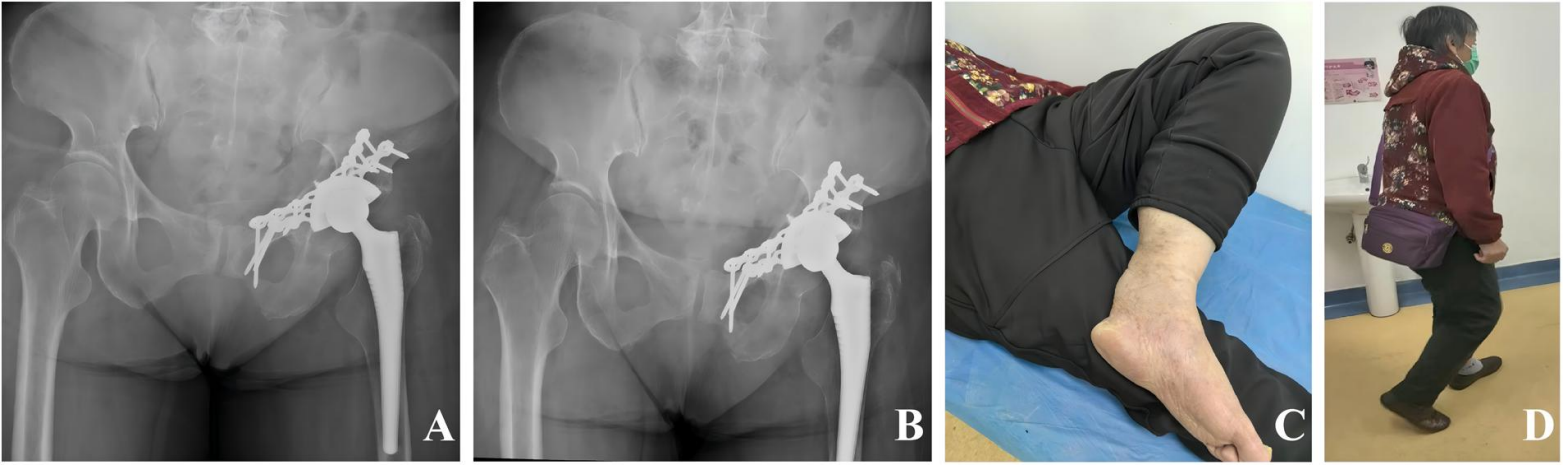
Postoperative follow-up images. **(A)** postoperative anteroposterior x-ray images of the pelvis at 1 month postoperatively. **(B)** postoperative anteroposterior x-ray images of the pelvis at 6 months postoperatively. **(C)** Image of hip flexion at 6 months after surgery. **(D)** Image of standing and walking at 6 months after surgery.

**Figure 4:**
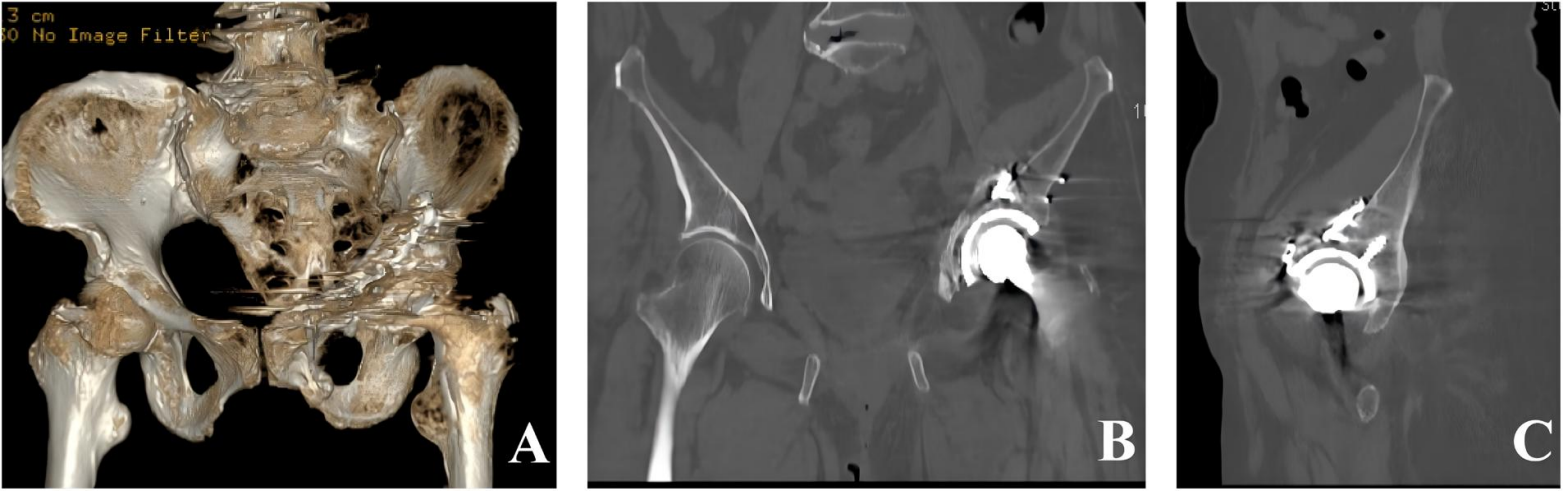
Postoperative CT images of the pelvis at 6 months postoperatively. **(A)** Three-dimensional reconstruction. **(B)** Coronal CT image. **(C)** Sagittal CT image.

The operative time was 160 min, and the estimated blood loss was 400 mL. Given the patient's advanced age and intraoperative blood loss, 3.5 units of packed red blood cells and 150 mL of plasma were transfused intraoperatively. Postoperative imaging demonstrated satisfactory reduction of the acetabular fracture and appropriate positioning of the plates and screws, with no evidence of screw penetration into the acetabular cup or the joint space (Figures 2D–G). Hip function was evaluated using the Harris Hip Score (HHS) during postoperative follow-up. Radiographs obtained at 1 and 3 months postoperatively confirmed stable positioning of both the acetabular component and the femoral stem (Figures 3A,B). The HHS was 42 and 80, respectively. At 6 months, the patient was able to perform activities of daily living, including hip and knee flexion, and ambulated independently (Figures 3C,D). The HHS was 89. Follow-up radiographs and CT demonstrated osseous union of the both-column acetabular fracture, with stable fixation and a well-fixed acetabular cup. Besides, no prosthesis-related loosening, dislocation, deep infection, or other complications were observed (Figure 4) (13).

### 2.5 Patient perspective

The patient reported progressive improvement in pain and confidence with mobilization during follow-up. The patient expressed satisfaction with avoiding revision surgery and preserving the existing hip arthroplasty.

## 3 Discussion

This case describes a rare and complex injury: a high-energy both-column acetabular fracture involving the quadrilateral surface in a patient with a prior THA. This represents a more complex subtype of traumatic PPAF and is particularly uncommon (14–16). Both-column acetabular fractures account for approximately 20% of acetabular fractures and are classically characterized as a “floating acetabulum,” making treatment highly challenging; the presence of an acetabular component further increases diagnostic and therapeutic complexity (17). In recent years, with the rising volume of THA and advances in trauma care, the recognition of PPAF and corresponding treatment algorithms have increasingly converged on a decision framework centered on cup stability (10–12, 18). When the acetabular component is unstable, revision arthroplasty combined with fracture fixation is typically required to restore load transfer and mitigate the risk of early failure (19). Conversely, when the cup is stable and osseointegrated, fixation aimed at restoring osseous support and pelvic continuity may allow cup retention and avoid the morbidity of revision surgery—an especially important consideration in older patients. Besides, Akbulut et al. reported that, in patients with well-bone-integrated and stable acetabular cups, periacetabular osteotomy can be considered a viable alternative for the treatment of recurrent instability caused by abnormal cup inclination (20). This is particularly applicable when the primary issue is a functional malposition of a stable cup rather than structural damage, and the cost of revision surgery is deemed excessively high.

Compared with primary acetabular fractures, PPAFs add the mechanical challenges of the implant–bone interface and compromised bone stock to the inherent difficulty of reducing an intra-articular fracture (21). In both-column fractures, disruption of pelvic ring continuity and the tendency toward medialization are more pronounced; inadequate reduction of the quadrilateral surface can result in acetabular protrusion and articular incongruity, thereby increasing the risk of delayed component loosening and the need for revision (10, 22). In our patient, the acetabular cup had not been completely impacted or displaced away from the medial osseous buttress, the fracture line did not extend into the greater sciatic notch, and the cup remained relatively stable (11). Meanwhile, the quadrilateral plate was osteopenic and prone to collapse and displacement after fracture. The presence of the implant also constrained the available corridors for fixation; excessively long screws may penetrate the acetabular shell or injure intrapelvic structures, further increasing operative risk (23). Prior biomechanical work suggests that when the host–implant contact area is <50%, adequate fixation strength cannot be achieved (24), and the remaining contact region is subjected to stress concentration that may exceed the mechanical tolerance of bone, promoting bone resorption and periprosthetic osteolysis and thereby accelerating loosening (14, 18). These considerations underscore the need for precise anatomic reduction and restoration of load-bearing support.

In this patient, preoperative CT raised concern for acetabular component instability due to circumferential disruption of the periacetabular bony ring. However, imaging may overestimate instability, particularly when fracture lines course adjacent to the cup without disrupting bone ingrowth at the implant–host interface. Therefore, intraoperative verification is essential. We confirmed stability through direct inspection of the cup–host bone interface and fluoroscopic assessment under gentle stress. Once stability was established, our operative objectives were to (i) restore the anatomy of both columns, (ii) reinforce medial support of the quadrilateral surface to prevent secondary medialization, and (iii) maintain safe screw trajectories around the existing acetabular component.

Given these requirements, we selected the lateral rectus abdominis approach, primarily for its advantages in complex acetabular fracture surgery. Compared with the traditional ilioinguinal approach, the lateral rectus abdominis approach typically uses a shorter incision (approximately 6–10 cm), accesses the pelvis through the extraperitoneal corridor, and avoids extensive dissection of the intricate inguinal neurovascular structures, thereby limiting surgical trauma and blood loss. This approach provides direct intrapelvic visualization of the anterior column, posterior column, and quadrilateral surface, facilitating reduction under direct vision and enabling buttress fixation of the quadrilateral plate—while avoiding the uncertainty inherent to indirect reduction techniques (25). Moreover, direct visualization allows real-time assessment of the relationship between the quadrilateral surface and the acetabular component and helps determine whether the cup is loose. In contrast to the pararectus approach, which involves opening the rectus sheath, the lateral rectus abdominis approach splits the external oblique aponeurosis. Because the muscle layers in this region are well vascularized, this corridor may offer favorable conditions for soft-tissue healing and is associated with less postoperative pain, shorter recovery, and a lower rate of soft-tissue–related complications (25). Beyond fracture union, when cup retention is planned, fixation must restore effective structural support of the acetabular dome and medial wall to prevent secondary cup migration under load. At the same time, it is necessary to protect the obturator nerve, peritoneum, and other surrounding structures during the operation, avoid direct impact of instruments on the acetabular cup, and reduce the risk of prosthesis wear or displacement.

Although the LRAA has been described for complex acetabular fractures, the contribution of this report is the periprosthetic context: a traumatic both-column acetabular fracture with quadrilateral surface medialization in the presence of a well-fixed, osteointegrated acetabular component. We emphasize a practical decision-making pathway for when cup retention is appropriate, how to achieve anatomic reduction and medial buttress support without jeopardizing the implant–bone interface, and how to monitor for delayed loosening. This aims to advance clinical applicability beyond prior LRAA series by focusing on reproducible intraoperative assessment of cup stability and fixation strategy in traumatic periprosthetic acetabular fractures.This provides a referable surgical paradigm for this rare but highly challenging fracture type.

Several limitations should be acknowledged. First, as a single-case report, the findings are inherently limited by the absence of large-cohort, long-term follow-up data, and the generalizability of the outcomes requires further validation. Second, the follow-up duration remains insufficient to assess long-term cup wear, late loosening, or the eventual need for revision.

Despite these limitations, this case demonstrates that a high-energy both-column acetabular fracture after THA can be successfully managed through the lateral rectus abdominis approach with cup retention, achieving reliable fracture union and satisfactory functional recovery. The key value of this strategy lies in leveraging a minimally invasive corridor that enables direct reduction and stable fixation, thereby addressing the technical challenges of this rare injury pattern while potentially reducing the risk of implant-related complications, including loosening. Besides, It provides a safe and reproducible criterion for the evaluation of intraoperative acetabular cup stability. In summary, open reduction and internal fixation of both-column acetabular fractures via the lateral rectus abdominis approach may represent an effective option for selected patients with high-energy PPAF after THA when the acetabular component is stable; however, further cases and longer follow-up are needed to refine operative workflows and fixation strategies and to provide more robust evidence.

## 4 Conclusion

Traumatic both-column acetabular fractures after THA are exceedingly rare and technically demanding to manage. Treatment decisions should be guided primarily by an assessment of acetabular cup stability. When intraoperative evaluation confirms a stable component, the lateral rectus abdominis approach can facilitate anatomic reduction of both columns and medial buttress fixation of the quadrilateral surface. This strategy preserves the acetabular component while restoring the load-bearing architecture of the acetabulum, thereby promoting fracture union and enabling satisfactory functional recovery.

## Data Availability

The datasets presented in this article are not readily available because of ethical and privacy restrictions. Requests to access the datasets should be directed to the corresponding authors.
